# Psychological and lifestyle risk factors for asthma exacerbations and morbidity in children

**DOI:** 10.1186/s40413-017-0169-9

**Published:** 2017-10-17

**Authors:** Alyssa A. Oland, Genery D. Booster, Bruce G. Bender

**Affiliations:** 1400 Jackson St, Denver, CO 80206 USA

**Keywords:** Asthma, Child, Risk, Adherence, Wellness, Treatment, Integrated care

## Abstract

Asthma is the most common childhood illness and disproportionately affects low-income, minority children who live in urban areas. A range of risk factors are associated with asthma morbidity and mortality, such as treatment non-adherence, exposure to environmental triggers, low-income households, exposure to chronic stress, child psychological problems, parental stress, family functioning, obesity, physical inactivity, and unhealthy diets. These risk factors often have complex interactions and inter-relationships. Comprehensive studies that explore the inter-relationships of these factors in accounting for asthma morbidity and mortality are needed and would help to inform clinical intervention. Considerable research has focused on interventions to improve adherence, asthma management, asthma symptoms, and quality of life for patients with asthma. Educational interventions combined with psychosocial interventions, such as behavioral, cognitive-behavioral, or family interventions, are beneficial and provide care in schools, homes, and emergency rooms can help to address barriers to accessing care for children and families. Additional recent research has explored the use of multidisciplinary, collaborative, integrated care with pediatric asthma patients, providing promising results. Integrated care could be ideal for addressing the multitude of complex psychosocial and wellness factors that play a role in childhood asthma, for increasing patient-centered care, and for promoting collaborative patient-provider relationships. Further research in this area is essential and would be beneficial.

## Background

Asthma is the most common chronic illness among children in the United States, affecting an estimated 10.6 million (14.5%) children [1], with significant impact on the physical and psychosocial well-being of affected youth. A child’s asthma can place significant stress and strain on parents and family systems. Considerable research has focused on childhood asthma, exploring disease risk factors, adherence, the role of child and caregiver psychological problems, the impact of family and environmental stressors, and the relationship between asthma and wellness factors such as obesity. Other research has also explored interventions to reduce adherence problems and improve overall functioning and asthma management in youth with asthma.

## Main text

### Environmental risk factors

Low-income, urban minority youth are disproportionately affected by asthma, such that higher asthma prevalence rates and disease morbidity have been found in children from low socioeconomic (SES) [2, 3] and urban minority families [3, 4]. Prevalence rates indicate that African American children have higher rates of asthma than Latino or White children [2], and Puerto Rican children have higher rates of asthma than other Latino children [3]. Additionally, African American and Latino children with asthma have more emergency room visits and higher mortality rates from asthma than White children with asthma [5]. Some research indicates that these racial differences in asthma prevalence rates are primarily present in low-income youth and not in minority children from middle- or upper- incomes [6].

Children living in low-income households often experience increased stress, such as family conflict, violence, lower quality home environments, dangerous neighborhoods, and polluted air and water [7], which is believed to be a risk factor for asthma [8]. Accordingly, housing-related stress [9], community stressors, such as high crime rates [10], poverty [11], chronic family stress [12], and exposure to tobacco smoke and pollutants [13] are associated with increased asthma symptoms. As one pathway to explain this relationship, exposure to chronic stress is associated with a heightened inflammatory profile that, in turn, is associated with increased asthma symptoms [12].

Additionally, research has found that children living in low-income households are more likely to experience reduced parental support and involvement, to spend more time watching television, and to feel more of a lack of control over their lives and living environments [7]. Children from economically disadvantaged households may also experience less access to care and poorer quality of care [14]; it is estimated that 41% of children aged 2–17 do not receive needed mental health services [1]. As such, low-income, urban minority children have increased barriers to proper asthma management, including increased exposure to environmental triggers and reduced access to appropriate treatment [8].

Further, lower parental education has been associated with higher prevalence rates of asthma, lower adherence with asthma medications, and higher rates of hospitalizations for asthma [15]. As parents in low-income households tend to have less education than parents in high-income households, this could also explain the relationship between low-income households and asthma.

### Adherence with asthma medication

It is well established that asthma medications, when taken properly, reduce asthma morbidity, emergency room visits, and hospitalizations, and poor adherence to asthma controller medications is associated with increased severe asthma exacerbations [16], uncontrolled asthma [17, 18], asthma morbidity [19, 20], and asthma mortality [21]. However, asthma controller medications tend to be underused in children [22] and medication non-adherence is a significant concern [23], especially among urban minority patients [22]. Given these results, interventions to improve medication adherence are important for reducing asthma morbidity and mortality. Additionally, improved asthma medication adherence and consequent improved asthma control can lead to increased quality of life [24].

Several risk factors appear to undermine medication adherence in children, including male gender, non-Asian ethnic background, living in a larger household, older age at diagnosis [25], living in rural areas [22], and lower socio-economic status [26]. Factors that contribute to non-adherence can generally be classified as intentional or non-intentional [27]. Unintentional factors include obstacles and barriers that result in medications not being taken by reasons other than one’s choice. Lack of parental involvement [28], lack of access to appropriate medications, improper inhaler technique [26], child psychological distress [29], adolescent forgetting [30], caregiver psychological distress, issues in family functioning [31], poor child and family understanding of asthma and asthma medications, limited symptom recognition, and a lack of community support [24]. Additionally, poor child and family understanding of asthma and asthma medications, limited symptom recognition, and a lack of community support can also contribute to medication non-adherence [24]. Intentional factors include beliefs about one’s illness and medication that result in a choice to not take medications as prescribed [32]. Intentional factors, such as parental concerns regarding controller medications (e.g., side effects, safety) and treatment cost [33], and adolescent beliefs that medication is not needed or beneficial [30] have been shown to be associated with higher rates of non-adherence.

### Psychological functioning in children with asthma and their caregivers

A majority of research suggests that children with asthma display more behavior problems [34] and internalizing disorders, such as anxiety and affective disorders, than their healthy peers [35]. The association between asthma and behavioral and emotional difficulties has been shown to significantly increase with asthma severity [34]. These psychological difficulties in children with asthma have been associated with increased functional limitations and poorer asthma management. For example, even after controlling for asthma severity, children with co-occurring asthma and internalizing disorder had increased functional impairments [35], more missed school days [36], increased use of rescue medications, poorer pulmonary functioning, more frequent emergent care use [37], and higher treatment non-adherence which, in turn, is associated with poorer health outcomes [29].

A child’s asthma can also affect caregiver adjustment, psychological functioning, and experience of stress. A significant increased risk for psychological difficulties in caregivers of children with asthma is reflected in increased rates of depression [29, 38] and anxiety [37]. These psychological difficulties in caregivers of children with asthma may contribute to problems in asthma management. For example, parental stress is significantly related to poorer medication adherence, and maternal depressive symptoms are associated with more problems in using proper inhaler technique, poorer medication adherence, greater exposure to tobacco smoke, and lower confidence in their abilities to manage their child’s asthma [39].

### Wellness and lifestyle

Recent research has focused on wellness factors, such as health promoting or health threatening lifestyle behaviors (e.g., diet, exercise), and their relevance to patients with asthma. Investigators have found a positive relationship between obesity and asthma in pediatric populations [40, 41]. Obesity in children and adolescents, in general, has also been associated with increased depression and anxiety [42, 43]. In children with asthma, obesity has been found to be associated with increased uncontrolled asthma [13, 44], asthma severity [41, 45], emergency care use, and corticosteroid use [46]. Additionally, obesity presents challenges to asthma management, such as decreased responsiveness to asthma controller medications and decreased quality of life [47], and overweight children with asthma have been found to have increased negative effects from pollutant exposure when compared to healthy-weight children with asthma [13].

Low levels of physical activity can be seen in children with asthma and contribute to obesity and psychological health problems. For example, children with asthma have been shown to have lower levels of physical activity [48, 49] and higher levels of engagement in sedentary activities, such as time spent on a computer [50]. Additionally, overweight children with asthma have been shown to be significantly less likely to engage in physical exercise than overweight children without asthma [51]. Increased inactivity can contribute to the problematic relationship between asthma and obesity. For example, in overweight children with asthma, increased time spent watching television was associated with increased risk of respiratory symptoms [52, 53] whereas increased physical activity was associated with reduced risk of respiratory symptoms [53]. Additionally, it is suggested that reduced physical activity results in increased asthma morbidity [54]. Further, data suggests that, for children with asthma, there is a positive relationship between level of physical activity and mental health [48].

Similarly, diet is related to weight and affects asthma. Children who mostly eat a “Western” diet, characterized by high amounts of fat and processed foods, have been shown to have higher asthma prevalence rates than children who eat diets with less fat and processed foods [55]. The Mediterranean diet has been associated with reduced asthma symptoms [56] and it is suggested that adherence to a healthy diet may have beneficial, protective effects on children’s asthma [57]. One possible explanation for the relationship between diet and asthma is the effect of diet on intenstinal microbiota and, in turn, the effect of intenstinal microbiota on immune inflammatory responses [58].

### Psychosocial interventions

There are a range of psychosocial interventions for children with asthma, such as educational programs, behavioral interventions, cognitive-behavioral therapy, family interventions, and/or community-based interventions. Research on the efficacy of psychosocial interventions is limited, inconclusive [59, 60], and plagued by concerns regarding methodological issues [60]. Despite these issues, preliminary evidence suggests that psychosocial interventions can improve the quality of life and medical outcomes for children with asthma. Interventions that combine education with psychosocial interventions, such as behavioral, cognitive-behavioral, and/or family interventions show particular promise [31].

Some interventions, such as self-management training and education, are considered well-established and essential parts of treatment for asthma patients throughout their course of care. Research on these programs has found benefits for children with asthma, including improved knowledge and confidence regarding disease management [61, 62], improved adherence [23, 63], improved asthma symptoms [64], improved lung function [65], reduced emergency room visits [65, 66], fewer school absences, less activity restriction [65], and improved quality of life [66]. However, there are considerable global disparities in regards to access to culturally-sensitive, age-appropriate patient education materials [67].

Given concerns regarding lack of access to effective interventions, particularly for children from disadvantaged backgrounds, some investigators have increasingly focused on exploring school-based, emergency-room based, and home-based educational interventions. When these interventions included parents, results indicated that parent self-efficacy for disease management improved [68]. Emergency room interventions are associated with increased adherence and reduced future emergency visits [69]. Further, research has found that home-based interventions reduce asthma symptoms and emergency visits [70, 71].

Despite these results, comprehensive meta-analyses of results across studies have concluded that education interventions, without additional interventions, are not sufficient for improving asthma management and health outcomes [72]. Investigations have shown that incorporating psychosocial interventions, such as behavioral or cognitive-behavioral interventions, into asthma education programs resulted in reduced asthma severity, reductions in emergency room visits [73], reduced child depression, reduced children stress [74], reduced school absences [73], improved adherence, and reduced parent–child conflict [75]. Similarly, education combined with family therapy resulted in benefits for children and for parents, resulting in reduced airway inflammation, improved physical health, and improved mental health. Parents also showed increased parental efficacy in regards to asthma management and reduced parental anxiety [76]. Additionally, one study found that increasing parental supervision increased child medication adherence [77].

For providers working with overweight pediatric patients, interventions to increase levels of physical activity and reduce unhealthy eating behaviors is emphasized [47]. A behavioral intervention focused on improving dietary intake, for example, resulted in reduced BMI and improved asthma [78] and an intervention that included psychological, nutritional, and exercise components showed reduced BMI, improved asthma control, and improved lung functioning [79].

### Integrated care

Given the complex array of factors influencing onset and severity of asthma, addressing this challenge requires bringing together multiple intervention components tailored to address the needs of individual children and their families. Recent evidence points to the importance of comprehensive, collaborative, multi-disciplinary care – known as *integrated care* - that addresses mental health, patient education, and family functioning in addition to traditional medical care [80]. Guidelines from the National Institute for Health [81], for example, specifically highlight the benefits of an interdisciplinary approach for patients with complicating psychological comorbidities, psychosocial stressors, and treatment non-adherence. Worldwide, institutions are working to develop integrated care through comprehensive care pathways for the treatment of asthma [82]. As such, a comprehensive approach to teaching asthma self-management skills that includes involvement of the community in program design and significantly focuses on family stress, family relationships [83, 84], family conflict, parental stress, parenting styles, and child behavior [84] may produce greater benefit and outcomes for children with asthma.

Integrated care programs that provide integrated care have demonstrated significant improvements in asthma symptoms, perceived competence in asthma management, reduced corticosteroid use, and improved quality of life for caregivers and children [85, 86]. In particular, engagement interventions that focus on specifically addressing barriers to treatment, such as telephone contacts, inclusion of brief interventions, and parent problem-solving, can significantly increase attendance at initial appointments and continued participation in treatment [87]. These strategies have shown promising effectiveness for adolescent substance abuse [88, 89], child mental health [90], and attention deficit/hyperactivity disorder [91].

Integrated care can occur in multiple settings according to level of patient need. Pediatric psychologists are often employed in inpatient settings or in tertiary care clinics as a part of the consultation-liaison services [92], but are increasingly incorporated into integrated care teams [80, 85]. Behavioral health practitioners can help medical providers implement adherence promotion interventions during medical appointments [93]. Finally, a movement toward early intervention and preventative care [94, 95] offers opportunities for multidisciplinary interventions within primary care settings [96] and school-based health centers [97, 98]. As children spend a significant proportion of their day at school (often 6–8 h), schools offer a unique opportunity for medication administration, parental support, and behavioral intervention. Studies of school-based interventions have found benefits that include improved knowledge, self-efficacy, and disease management [62] and improved asthma control [99].

## Conclusions

**Fig. 1:**
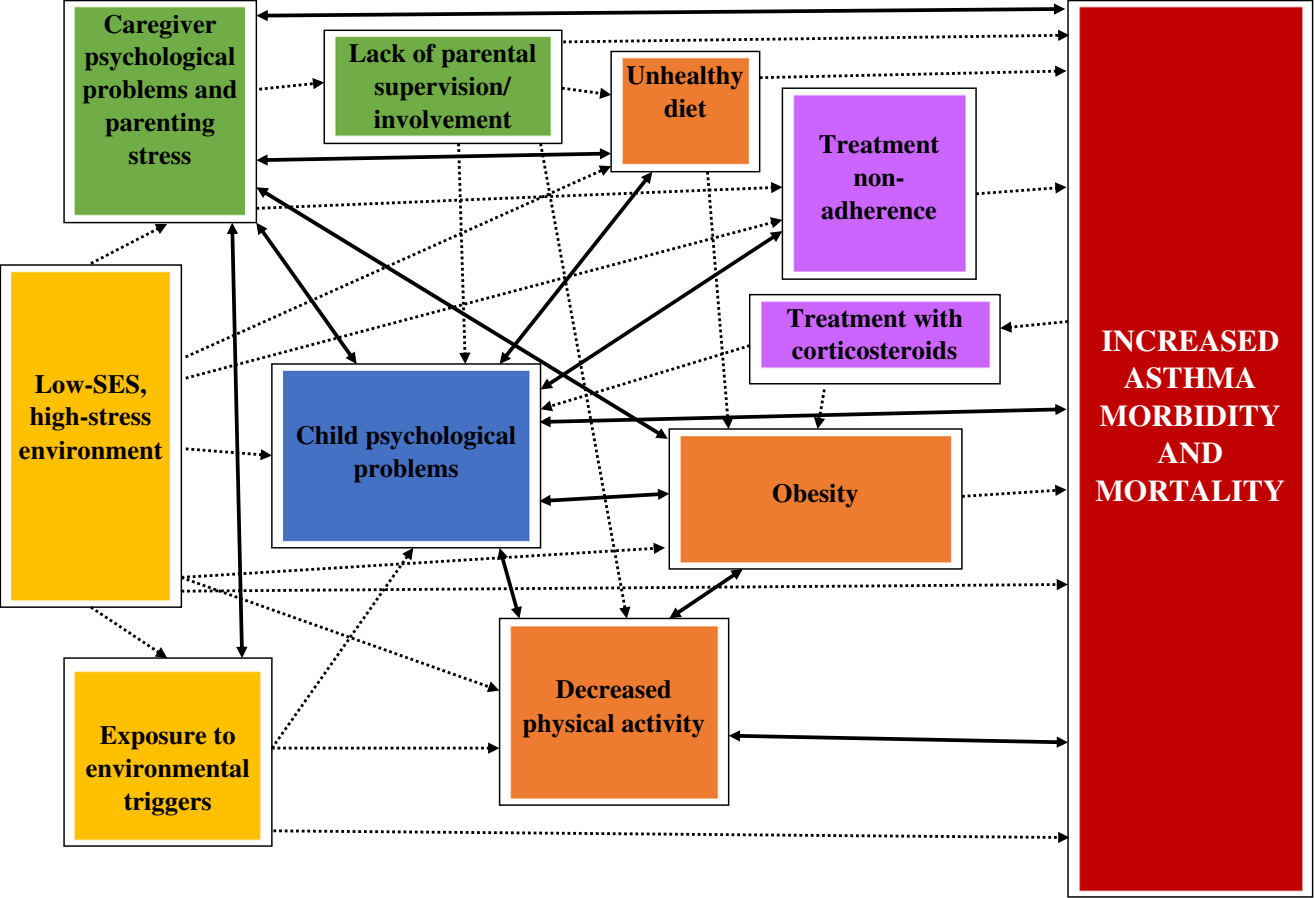
Psychosocial and lifestyle risk factors in pediatric asthma

A range of environmental, psychosocial, behavioral, and lifestyle risk factors are associated with asthma exacerbations and morbidity. These risk factors have complex interactions and bidirectional relationships (see Fig. 1). The vast majority of research has focused on one or a few of these factors in isolation. Despite this often singular focus, it is not uncommon for several risk factors to emerge in research studies, such that there is often some overlap in findings across studies regardless of initial focus. For example, studies of child risk factors often find parenting factors to be relevant. Given the complex interactions between risk factors, comprehensive studies that explore the inter-relationships of all or most of these factors in accounting for asthma morbidity and mortality are needed and would help to inform clinical intervention.

Accordingly, comprehensive approaches to treatment that consider the interplay between personal, familial, and environmental risk factors are needed. Recent research has underscored the importance of multidisciplinary, collaborative, integrated care for pediatric asthma patients with promising results. Such a comprehensive approach is ideal for addressing the multitude of complex, inter-related psychosocial and wellness/lifestyle factors that play a role in childhood asthma. This integrated care approach could also be productive for increasing patient-centered care, shared decision making, and collaborative relationships between families and providers. Further research in this area is needed and would be beneficial.

## References

[1] U.S. Department of Health and Human Services, Health Resources and Services Administration, Maternal and Child Health Bureau, The Health and Well-Being of Children: A Portrait of States and the Nation, 2011-2012.Rockville, Maryland: U.S. Department of Health and Human Services; 2014.

[2] Centers for Disease Control and Prevention (2011). Vital signs: asthma prevalence, disease characteristics, and self-management education: United States, 2001–2009. MMWR Morb Mortal Wkly Rep.

[3] Gold DR, Wright R. Population disparities in asthma. Annu Rev Public Health. 2005; doi:10.1146/annurev.publhealth.26.021304.144528. 10.1146/annurev.publhealth.26.021304.144528 15760282

[4] Bloom B, Cohen RA (2007). Summary health statistics for U.S. children: National Health Interview Survey, 2006. Vital Health Stat 10.

[5] Moorman JE, Akinbami LJ, Bailey CM, Zahran HS, King ME, Johnson CA, Liu X (2012). National surveillance of asthma: United States, 2001–2010. Vital Health Stat 3.

[6] Smith LA, Hatcher-Ross JL, Wertheimer R, Kahn RS. Rethinking race/ethnicity, income, and childhood asthma: racial/ethnic disparities concentrated among the very poor. Public Health Rep. 2005; 10.1177/003335490512000203 PMC1497701 15842111

[7] Adler NE, Conner SA (2003). The role of psychosocial processes in explaining the gradient between socioeconomic status and health. Curr Dir Psychol Sci.

[8] Basch CE. Asthma and the achievement gap among urban minority youth. J Sch Health. 2011; 10.1111/j.1746-1561.2011.00634.x 21923872

[9] Sandel M, Wright RJ. When home is where the stress is: expanding the dimensions of housing that influence asthma morbidity. Arch Dis Child. 2006; doi:10.1136/adc.2006.098376. 10.1136/adc.2006.098376 PMC2082962 17056870

[10] Wright RJ. Health effects of socially toxic neighborhoods; the violence and urban asthma paradigm. Clin Chest Med. 2006; doi:10.1016/j.ccm.2006.04.003. 10.1016/j.ccm.2006.04.003 16880051

[11] Chen E, Fisher E, Bacharier LB, Strunk RC. Socioeconomic status, stress, and immune markers in adolescents with asthma. Psychosom Med. 2003; doi:10.1097/01.PSY.0000097340.54195.3C. 10.1097/01.psy.0000097340.54195.3c 14645776

[12] Marin TJ, Chen E, Munch JA, Miller GE. Double-exposure to acute stress and chronic family stress is associated with immune changes in children with asthma. Psychosom Med. 2009; doi:10.1097/PSY.0b013e318199dbc3. 10.1097/PSY.0b013e318199dbc3 PMC2735247 19196805

[13] Sheehan WJ, Philatanakul W. Difficult-to-control asthma: epidemiology and its link with environmental factors. Curr Opin Allergy Clin Immunol. 2015; doi:10.1097/ACI.0000000000000195. 10.1097/ACI.0000000000000195 PMC4551573 26226354

[14] U.S. Department of Health and Human Services, Agency for Healthcare Research and Quality, National Healthcare Disparities Report, 2012. Rockville, Maryland: U.S. Department of Health and Human Services; 2013.

[15] Gong T, Lundholm C, Rejno G, Mood C, Langstrom N, Almqvist C. Parental socioeconomic status, childhood asthma and medication use--a population-based study. PLoS One. 2014; doi:10.1371/journal.pone.0106579. 10.1371/journal.pone.0106579 PMC4154738 25188036

[16] Engelkes M, Janssens HM, de Jongste JC, Sturkenboom MC, Verhamme KM. Medication adherence and the risk of severe asthma exacerbations: a systematic review. Eur Respir J. 2015; doi:10.1183/09031936.00075614. 10.1183/09031936.00075614 25323234

[17] Blake KV. Improving adherence to asthma medications: current knowledge and future perspectives. Curr Opin Pulm Med. 2017; doi:10.1097/MCP.0000000000000334. 10.1097/MCP.0000000000000334 27755160

[18] Herndon JB, Mattke S, Evans Cuellar A, Hong SY, Shenkman EA. Anti-inflammatory medication adherence, healthcare utilization and expenditures among Medicaid and Children's health insurance program enrollees with asthma. PharmacoEconomics. 2012; doi:10.2165/11586660-000000000-00000. 10.2165/11586660-000000000-00000 22268444

[19] Bauman LJ, Wright E, Leickly EE (2002). Relationship of adherence to pediatric asthma morbidity among inner-city children. Pediatrics.

[20] McQuaid EL, Kopel SJ, Lkein RB, Fritz GK (2003). Medication adherence in pediatric asthma: reasoning, responsibility, and behavior. J Pediatr Psychol.

[21] Suissa S, Ernst P, Benayoun S, Balzan M, Cai B. Low-dose inhaled corticosteroids and the prevention of death from asthma. N Engl J Med. 2000; doi:10.1056/NEJM200008033430504. 10.1056/NEJM200008033430504 10922423

[22] Halterman JS, Auinger P, Conn KM, Lynch K, Yoos HJ, Szilagyi PG. Inadequate therapy and poor symptom control among children with asthma: findings from a multistate sample. Ambul Pediatr. 2007; doi:10.1016/j.ambp.2006.11.007. 10.1016/j.ambp.2006.11.007 17368410

[23] Morton RW, Everard ML, Elphick HE. Adherence in childhood asthma: the elephant in the room. Arch Dis Child. 2014; doi:10.1136/archdischild-2014-306243. 10.1136/archdischild-2014-306243 24876303

[24] Friend M, Morrison A. Interventions to improve asthma Management of the School-age Child. Clin Pediatr (Phila). 2015; doi:10.1177/0009922814554500. 10.1177/0009922814554500 25320062

[25] Chan AHY, Stewart AW, Foster JM, Mitchell EA, Camargo CA, Harrison J. Factors associated with medication adherence in school-aged children with asthma. ERJ Open Res. 2016;2:1–9. 10.1183/23120541.00087-2015 PMC5005164 27730181

[26] Pappalardo AA, Karavolos K, Martin MA. What really happens in the home: the medication environment of urban, minority youth. J Allergy Clin Immunol Pract. 2016;5(3):764–770. 10.1016/j.jaip.2016.09.046 PMC5423821 27914817

[27] Wroe AL. Intentional and unintentional nonadherence: a study of decision making. J Behav Med. 2002; doi:10.1023/A:1015866415552. 10.1023/a:1015866415552 12136497

[28] Warman K, Silver EJ, Wood PR. Asthma risk factor assessment: What are the needs of inner city families? Ann Allergy Asthma Immunol. 2006;97Suppl:S11-S15. 10.1016/s1081-1206(10)60779-x 16892765

[29] Bender B. Risk taking, depression, adherence, and symptom control in adolescents and young adults with asthma. Am J Respir Crit Care Med. 2006; doi:10.1164/rccm.200511-1706PP. 10.1164/rccm.200511-1706PP 16424441

[30] Koster ES, Philbert D, de Vries TW, van Dijk L, Bouyy ML. I just forget to take it: asthma self-management needs and preferences in adolescents. J Asthma. 2015; doi:10.3109/02770903.2015.1020388. 10.3109/02770903.2015.1020388 26037397

[31] Booster G, Oland A, Bender B. Psychosocial factors in severe pediatric asthma. Immunol Allergy Clin N Am. 2016; doi:10.1016/j.iac.2016.03.012. 10.1016/j.iac.2016.03.012 27401618

[32] Kolk T, Kaptein AA, Brand PL. Non-adherence in children with asthma reviewed: the need for improvement of asthma care and medical education. Pediatr Allergy Immunol. 2015; doi:10.1111/pai.12362. 10.1111/pai.12362 25704083

[33] Mirsadraee R, Gharagozlou M, Movahedi M, Behniafard N, Nasiri R (2012). Evaluation of factors contributed in nonadherence to medication therapy in children asthma. Iran J Allergy Asthma Immunol.

[34] McQuaid EL, Kopel SJ, Nassau JH (2001). Behavioral adjustment in children with asthma: a meta-analysis. J Dev Behav Pediatr.

[35] Katon W, Lozano P, Russo J, McCauley E, Richardson L, Bush T. The prevalence of DSM-IV anxiety and depressive disorders in youth with asthma compared with controls. J Adolesc Health. 2007; doi:10.1016/j.jadohealth.2007.05.023. 10.1016/j.jadohealth.2007.05.023 PMC2153460 17950165

[36] Bender B, Zhang L. Negative affect, medication adherence, and asthma control in children. J Allergy Clin Immunol. 2008; doi:10.1016/j.jaci.2008.05.041. 10.1016/j.jaci.2008.05.041 18602153

[37] Feldman JM, Steinberg D, Kutner H, Eisenberg N, Hottinger K, Sidora-Arcoleo K, Warman K, Serebrisky D. Perception of pulmonary function and asthma control: the differential role of child versus caregiver anxiety and depression. J Pediatr Psychol. 2013; doi:10.1093/jpepsy/jsto52. 10.1093/jpepsy/jst052 PMC3809726 23873703

[38] Easter G, Sharpe L, Hunt CJ. Systematic review and meta-analysis of anxious and depressive symptoms in caregivers of children with asthma. J Pediatr Psychol. 2015; doi:10.1093/jpepsy/jsv012. 10.1093/jpepsy/jsv012 25829528

[39] Lim JH, Wood BL, Miller BD, Simmens SJ. Effects of paternal and maternal depressive symptoms on child internalizing symptoms and asthma disease activity: mediation by interparental negativity and parenting. J Fam Psychol. 2011; doi:10.1037/a0022452. 10.1037/a0022452 PMC3061476 21355653

[40] Liu P, Kieckhefer GM, Gau B. A systematic review of the association between obesity and asthma in children. J Adv Nurs. 2013; doi:10.1111/jan.12129. 10.1111/jan.12129 PMC3723337 23560878

[41] Michelson PH, Williams LW, Benjamin DK, Barnato AE. Obesity, inflammation and asthma severity in childhood: data from the National Health and nutrition examination survey 2001–2004. Ann Allergy Asthma Immunol. 2009; doi:10.1016/S1081-1206(10)60356-0. 10.1016/S1081-1206(10)60356-0 19927535

[42] Herget S, Rudolph A, Hilbert A, Blüher S. Psychosocial status and mental health in adolescents before and after bariatric surgery: a systematic literature review. Obes Facts. 2014; doi:10.1159/0000365793. 10.1159/000365793 PMC5644788 25059420

[43] Hasler G, Gergen PJ, Ajdacic V, Gamma A, Eich D, Rössler W, Angst J. Asthma and body weight change: a 20-year prospective community study of young adults. Int J Obes. 2006; doi:10.1038/sj.ijo.0803215. 10.1038/sj.ijo.0803215 16491113

[44] Ferreira-Magalhães M, Pereira AM, Sa-Sousa A, Morais-Almeida M, Azevedo I, Azevedo LF, Fonseca JA. Asthma control in children is associated with nasal symptoms, obesity, and health insurance: a nationwide survey. Pediatr Allergy Immunol. 2015; doi:10.1111/pai.12395. 10.1111/pai.12395 25939454

[45] Hacihamdioglu B, Arslan M, Yeşilkaya E, Gok F, Yavuz ST. Wider neck circumference is related to severe asthma in children. Pediatr Allergy Immunol. 2015; doi:10.1111/pai.12402. 10.1111/pai.12402 25952270

[46] Black M, Smith N, Porter A, Jacobsen S, Koebnick C. Higher prevalence of obesity among children with asthma. Obesity. 2012; doi:10.1038/oby.2012.5. 10.1038/oby.2012.5 PMC3348709 22252049

[47] Lang J. Obesity and asthma in children: current and future therapeutic options. Pediatr Drugs. 2014; 10.1007/s40272-014-0069-1 PMC4355012 24604125

[48] Glazebrook C, McPherson AC, Macdonald IA, Swift JA, Ramsay C, Newbould R, Smyth A. Asthma as a barrier to children’s physical activity: implications for body mass index and mental health. Pediatrics. 2006; doi:10.1542/peds.2006-1846. 10.1542/peds.2006-1846 17142530

[49] Lam K, Yang Y, Wang L, Chen S, Gau B, Chiang B. Original article: physical activity in school-aged children with asthma in an Urban City of Taiwan. Pediatr Neonatol. 2015;57(4):333–337. 10.1016/j.pedneo.2015.05.003 27118301

[50] Jones S, Merkle S, Fulton J, Wheeler L, Mannino D (2006). Relationship between asthma, overweight, and physical activity among U.S. high school students. J Community Health.

[51] Lawson JA, Rennie DC, Dosman JA, Cammer AL, Senthilselvan A. Obesity, diet, and activity in relation to asthma and wheeze among rural dwelling children and adolescents. J Of Obesity. 2013; doi:10.1155/2013/315096. 10.1155/2013/315096 PMC3804370 24191194

[52] Mitchell EA, Beasley R, Björkstén B, Crane J, García-Marcos L, Keil U. The association between BMI, vigorous physical activity and television viewing and the risk of symptoms of asthma, rhinoconjunctivitis and eczema in children and adolescents: ISAAC phase three. Clin Exp Allergy. 2013; doi:10.1111/cea.12024. 10.1111/cea.12024 23278882

[53] Tsai H, Tsai AC, Nriagu J, Ghosh D, Gong M, Sandretto A (2007). Associations of BMI, TV-watching time, and physical activity on respiratory symptoms and asthma in 5th grade schoolchildren in Taipei Taiwan. J Asthma.

[54] Lucas SR, Platts-Mills TA (2005). Physical activity and exercise in asthma: relevance to etiology and treatment. J Allergy Clin Immunol.

[55] Patel S, Custovic A, Smith JA, Simpson A, Kerry G, Murray CS. Cross-sectional association of dietary patterns with asthma and atopic sensitization in childhood - in a cohort study. Pediatr Allergy Immunol. 2014; doi:10.1111/pai.12276. 10.1111/pai.12276 25201630

[56] Alphantonogeorgos G, Panagiotakos DB, Grigoropoulou D, Yfanti K, Papoutsakis C, Papadimitriou A, Anthracopoulos MB, Bakoula C, Priftis KN (2014). Investigating the associations between Mediterranean diet, physical activity and living environment with childhood asthma using path analysis. Endocr Metab Immune Disord Drug Targets.

[57] Saadeh D, Salameh P, Caillaud D, Charpin D, De Blay F, Kopferschmitt C, Lavaud F, Annesi-Maesano I, Baldi I, Raherison C. Prevalence and association of asthma and allergic sensitization with dietary factors in schoolchildren: data from the french six cities study. BMC Public Health. 2015; doi:10.1186/s12889-015-2320-2. 10.1186/s12889-015-2320-2 PMC4589972 26423141

[58] Maslowski D, Kackay C (2011). Nature immunology.

[59] Eccleston C, Palermo TM, Fisher E, Law E. Psychological interventions for parents of children and adolescents with chronic illness. Cochrane Database Syst Rev. 2015; doi:10.1002/14651858.CD009660.pub3. 10.1002/14651858.CD009660.pub4 PMC6450193 30883665

[60] Yorke J, Fleming SL, Shuldham C. A systematic review of psychological interventions for children with asthma. Pediatr Pulmonol. 2007; doi:10.1002/ppul.20464. 10.1002/ppul.20464 17186533

[61] Boyd M, Lasserson TJ, Mckean MC, Gibson PG, Ducharme FM, Haby M. Interventions for educating children who are at risk of asthma-related emergency department attendance. Cochrane Database Syst Rev. 2009; doi:10.1002/14651858.CD001290.pub2. 10.1002/14651858.CD001290.pub2 PMC7079713 19370563

[62] Coffman JM, Cabana MD, Yelin EH. Do school-based asthma education programs improve self-management and health outcomes? Pediatrics. 2009; doi:10.1542/peds.2008-2085. 10.1542/peds.2008-2085 PMC2875148 19651589

[63] Otsuki M, Eakin MN, Rand CS, Butz AM, Hsu VD, Zuckerman IH, Ogborn J, Bilderback A, Riekert KA. Adherence feedback to improve asthma outcomes among inner-city children: a randomized trial. Pediatrics. 2009; doi:10.1542/peds.2008-2961. 10.1542/peds.2008-2961 PMC5450495 19948623

[64] Georgiou A, Buchner DA, Ershoff DH, Blasko KM, Goodman LV, Feigin J. The impact of a large-scale population-based asthma management program on pediatric asthma patients and their caregivers. Ann Allergy Asthma Immunol. 2003; doi:10.1016/S1081-1206(10)61799-1. 10.1016/S1081-1206(10)61799-1 12669894

[65] Guevara JP, Wolf FM, Grum CM, Clark NM. Effects of educational interventions for self management of asthma in children and adolescents: systematic review and meta-analysis. BMJ. 2003; doi:10.1136/bmj.326.7402.1308. 10.1136/bmj.326.7402.1308 PMC161636 12805167

[66] Watson WT, Gillespie C, Thomas N, Filuk SE, McColm J, Piwniuk MP, Becker AB. Small-group, interactive education and the effect on asthma control by children and their families. CMAJ. 2009; doi:10.1503/cmaj.080947. 10.1503/cmaj.080947 PMC2734203 19687105

[67] Everard ML, Wahn U, Dorsano S, Hossny E, LeSouef P (2015). Asthma education material for children and their families; a global survey of current resources. World Allergy Organ J.

[68] Terpstra JL, Chavez LJ, Ayala GX. An intervention to increase caregiver support for asthma management in middle school-aged youth. J Asthma. 2012; doi:10.3109/02770903.2012.656866. 10.3109/02770903.2012.656866 22316141

[69] Teach SJ, Crain EF, Quint DM, Hylan ML, Joseph JG. Improved asthma outcomes in a high-morbidity pediatric population: results of an emergency department-based randomized clinical trial. Arch Pediatr Adolesc Med. 2006; doi:10.1001/archpedi.160.5.535. 10.1001/archpedi.160.5.535 16651498

[70] Brown JV, Bakeman R, Celano MP, Demi AS, Kobrynski L, Wilson SR. Home-based asthma education of young low-income children and their families. J Pediatr Psychol. 27(8):2002, 677–88. 10.1093/jpepsy/27.8.677 12403858

[71] Canino G, Vila D, Normand S-LT, Acosta-Perez E, Ramirez R, Garcia P, Rand C. Reducing asthma health disparities in poor Puerto Rican children: the effectiveness of a culturally tailored family intervention. J Allergy Clin Immunol. 2008; doi:10.1016/j.jaci.2007.10.022. 10.1016/j.jaci.2007.10.022 PMC3136215 18061648

[72] Clark SA, Calam R. The effectiveness of psychosocial interventions designed to improve health-related quality of life (HRQOL) amongst asthmatic children and their families: a systemic review. Qual Life Res. 2012; doi:10.1007/s11136-011-9996-2. 10.1007/s11136-011-9996-2 21901377

[73] Chen SH, Huang JL, Yeh KW, Tsai YF. Interactive support interventions for caregivers of asthmatic children. J Asthma. 2013; doi:10.3109/02770903.2013.794236. 10.3109/02770903.2013.794236 23586594

[74] Long KA, Ewing LJ, Cohen S, Skoner D, Gentile D, Koehrsen J, Howe C, Thompson AL, Rosen RK, Ganley M, Marsland AL. Preliminary evidence for the feasibility of a stress management intervention for 7- to 12-year-olds with asthma. J Asthma. 2011; doi:10.3109/02770903.2011.554941. 10.3109/02770903.2011.554941 21332379

[75] Duncan CL, Hogan MB, Tien KJ, Graves MM, Chorney JL, Zettler MD, Koven L, Wilson NW, Dinakar C, Portnoy J. Efficacy of a parent-youth teamwork intervention to promote adherence in pediatric asthma. J Pediatr Psychol. 2013; doi:10.1093/jpepsy/jss123. 10.1093/jpepsy/jss123 PMC3701124 23248342

[76] Ng SM, Li AM, Lou VW, Tso IF, Wan PY, Chan DF. Incorporating family therapy into asthma group intervention: a randomized waitlist-controlled trial. Fam Process. 2008; doi:10.1111/j.1545-5300.2008.00242.x. 10.1111/j.1545-5300.2008.00242.x 18411833

[77] Park G, Han HW, Kim HS, Kim JY, Lee E, Cho HJ, Yang SI, Jung YH, Hong SJ, Kim HY, Seo JH, Yu J. High degree of supervision improves adherence to inhaled corticosteroids in children with asthma. Korean J Pediatr, 2015 58(12):472-477. 10.3345/kjp.2015.58.12.472 PMC4705327 26770222

[78] Jensen ME, Gibson PG, Collins CE, Hilton JM, Wood LG. Dietinduced weight loss in obese children with asthma: a randomized controlled trial. Clin Exp Allergy. 2013; doi:10.1111/cea.12115. 10.1111/cea.12115 23786284

[79] da Silva PL, de Mello MT, Cheik NC, Sanches PL, Correia FA, de Piano A, Corgosinho FC, Campos RM, do Nascimento CM, Oyama LM, Tock L, Tufik S, Damaso AR. Interdisciplinary therapy improves biomarkers profile and lung function in asthmatic obese adolescents. Pediatr Pulmonol. 2012. doi:10.1002/ppul.21502. 10.1002/ppul.21502 22170805

[80] McQuaid EL, Fedele DA. Pediatric asthma. In: Roberts MC, Steele RG, editors. Handbook of pediatric psychology. fifth ed. New York: The Guilford Press; 2017. p. 227–40.

[81] National Asthma Education and Prevention Program, Third Expert Panel on the Diagnosis and Management of Asthma. Expert Panel Report 3: Guidelines for the Diagnosis and Management of Asthma. Bethesda (MD): National Heart, Lung, and Blood Institute (US); 2007. Available from: https://www.ncbi.nlm.nih.gov/books/NBK7232/.

[82] Bosquet J, Addis A, Adcock I (2014). Integrated care pathways for airway diseases (AIRWAYS-ICPs). Eur Respir J.

[83] Celano MP. Family processes in pediatric asthma. Curr Opin Pediatr. 2006; doi:10.1097/01.mop.0000245355.60583.74. 10.1097/01.mop.0000245355.60583.74 16969169

[84] Clarke SA, Calam R. The effectiveness of psychosocial interventions designed to improve health-related quality of life (HRQOL) amongst asthmatic children and their families: a systematic review. Qual Life Res. 2012; doi:10.1007/s11136-011-9996-2. 10.1007/s11136-011-9996-2 21901377

[85] Bratton DL, Price M, Gavin L, Glenn K, Brenner M, Gelfand EW, Klinnert MD (2001). Impact of a multidisciplinary day program on disease and healthcare costs in children and adolescents with severe asthma: a two-year follow-up study. Pediatr Pulmonol.

[86] Janevic MR, Stoll S, Wilkin M, Song PXK, Baptist A, Lara M, Ramos-Valencia G, Bryant-Stephens T, Persky V, Uyeda K, Lesch JK, Wang W, Malveaux FJ. Pediatric asthma care coordination in underserved communities: a quasiexperimental study. Am J Public Health. 2016; doi:10.2105/AJPH.2016.303373. 10.2105/AJPH.2016.303373 PMC5055769 27631740

[87] McKay MM, Bannon WM. Engaging families in child mental health services. Child Adolesc Psychiatr Clin N Am. 2004; doi:10.1016/j.chc.2004.04.001. 10.1016/j.chc.2004.04.001 15380788

[88] Coatsworth JD, Santisteban DA, McBride CK, Szapocznik J (2001). Brief strategic family therapy versus community control: engagement, retention, and an exploration of the moderating role of adolescent symptom severity. Fam Proess.

[89] Santisteban DA, Szapocznik J, Perez-vidal A, Kurtines WM, Murray EJ, LaPerriere A. Efficacy of intervention for engaging youth and families into treatment and some variables that may contribute to differential effectiveness. J Fam Psychol. 1996; doi:10.1037//0893-3200.10.1.35.

[90] McKay MM, McCadam K, Gonzales J. Addressing the barriers to mental health services for inner city children and their caretakers. Community Ment Health J. 1996; doi:10.1007/BF02249453. 10.1007/BF02249453 8840078

[91] Power TJ, Mautone JA, Marshall SA, Jones HA, Cacia J, Tresco K, Cassano MC, Jawad AF, Guevara JP, Blum NJ. Feasibility and potential effectiveness of integrated services for children with ADHD in urban primary care practices. Clin Pract Pediatr Psychol. 2014;2:421–426.

[92] Carter BD, Kronenberger WG, Scott EL, Kronenberger KA, Piazza-Waggoner C, Brady CW. Inpatient pediatric consultation-liaison. In: Roberts MC, Steele RG, editors. Handbook of pediatric psychology. fifth ed. New York: The Guilford Press; 2017. p. 105–18.

[93] Rohan JM, Drotar D, Perry AR, McDowell K, Malkin J, Kercsmar C. Training health care providers to conduct adherence promotion in pediatric settings: an example with pediatric asthma. Clin Pract Pediatr Psychol. 2013;1:314–325.

[94] Rawal P, MA MC (2016). Health care reform and programs that provide opportunities to promote children’s behavioral health.

[95] Rittenhouse DR, Shortell SM, Fisher ES. Primary care and accountable care – two essential elements of delivery-system reform. N Engl J Med. 2009; doi:10.1056/NEJMp0909327. 10.1056/NEJMp0909327 19864649

[96] Stancin T, Perrin E. Psychologists and pediatricians: opportunities for collaboration in primary care. Am Psychol. 2014; doi:10.1037/a0036046. 10.1037/a0036046 24820683

[97] Bruzzese JM, Evans D, Kattan M. School-based asthma programs. J Allergy Clin Immunol. 2009; doi:10.1016/j.jaci.2009.05.040. 10.1016/j.jaci.2009.05.040 19615728

[98] Clayton S, Chin T, Blackburn S. Echeverria. Different setting, different care: Integrating prevention and clinical care in school-based health centers. Am J Public Health. 2010; doi:10.2105/AJPH.2009.186668. 10.2105/AJPH.2009.186668 PMC2920951 20634447

[99] Gerald LB, Mcclure LA, Mangan JM, Harrington KF, Gibson L, Erwin S, Atchinson J, Grad R. Increasing adherence to inhaled steroid therapy among schoolchildren: randomized, controlled trial of school-based supervised asthma therapy. Pediatrics. 2009; doi:10.1542/peds.2008-0499. 10.1542/peds.2008-0499 PMC2782792 19171611

